# *trans*-Bis[4-amino-*N*-(pyrimidin-2-yl)benzene­sulfonamidato]dipyridine­cobalt(II) hemihydrate

**DOI:** 10.1107/S1600536810013802

**Published:** 2010-04-21

**Authors:** Yan-Fei Wang, Hong-Li Zou, Xu-Jian Luo, Zhen-Feng Chen, Hong Liang

**Affiliations:** a School of Chemistry and Chemical Engineering, Central South University, Changsha 410083, People’s Republic of China; b Key Laboratory for the Chemistry and Molecular Engineering of Medicinal Resources (Ministry of Education of China), School of Chemistry & Chemical Engineering, Guangxi Normal University, Guilin 541004, People’s Republic of China

## Abstract

The asymmeric unit of the title compound, [Co(C_10_H_9_N_4_O_2_S)_2_(C_5_H_5_N)_2_]·0.5H_2_O, contains the distorted octa­hedral *trans*-[Co(sdz)_2_(py)_2_] (sdz is the sulfadiazine anion and py is pyridine) complex mol­ecule and a half-mol­ecule of water, which lies on a twofold rotation axis. A three-dimensional network is generated by N—H⋯O and O—H⋯O hydrogen bonds between the complex and the water mol­ecules.

## Related literature

For mono ligand sulfadiazine–metal complexes, see: Yuan *et al.* (2001 ▶); Wang *et al.* (2005 ▶). For mixed ligand sulfadiazine–metal complexes, see: Ajibade *et al.* (2006 ▶); Brown *et al.* (1987 ▶); Hossain *et al.* (2006 ▶); Wang *et al.* (2009 ▶).
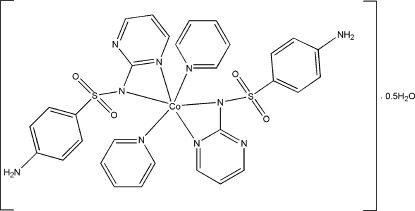


## Experimental

### 

#### Crystal data

[Co(C_10_H_9_N_4_O_2_S)_2_(C_5_H_5_N)_2_]·0.5H_2_O
*M* *_r_* = 724.68
Monoclinic, 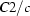

*a* = 39.618 (4) Å
*b* = 11.2407 (9) Å
*c* = 14.5673 (13) Å
β = 104.648 (2)°
*V* = 6276.4 (10) Å^3^
*Z* = 8
Mo *K*α radiation
μ = 0.74 mm^−1^
*T* = 193 K
0.44 × 0.15 × 0.12 mm

#### Data collection

Rigaku Mercury diffractometer
Absorption correction: multi-scan (*REQAB*; Jacobson, 1998 ▶) *T*_min_ = 0.738, *T*_max_ = 0.917
34520 measured reflections
7188 independent reflections
6038 reflections with *I* > 2σ(*I*)
*R*_int_ = 0.051

#### Refinement

*R*[*F* ^2^ > 2σ(*F* ^2^)] = 0.055
*wR*(*F* ^2^) = 0.103
*S* = 1.18
7188 reflections
434 parameters
H atoms treated by a mixture of independent and constrained refinement
Δρ_max_ = 0.36 e Å^−3^
Δρ_min_ = −0.52 e Å^−3^

### 

Data collection: *CrystalClear* (Rigaku, 1999 ▶); cell refinement: *CrystalClear*; data reduction: *CrystalStructure* (Rigaku/MSC & Rigaku, 2000 ▶); program(s) used to solve structure: *SHELXS97* (Sheldrick, 2008 ▶); program(s) used to refine structure: *SHELXL97* (Sheldrick, 2008 ▶); molecular graphics: *SHELXTL* (Sheldrick, 2008 ▶); software used to prepare material for publication: *SHELXTL*.

## Supplementary Material

Crystal structure: contains datablocks I, global. DOI: 10.1107/S1600536810013802/pk2239sup1.cif

Structure factors: contains datablocks I. DOI: 10.1107/S1600536810013802/pk2239Isup2.hkl

Additional supplementary materials:  crystallographic information; 3D view; checkCIF report

## Figures and Tables

**Table 1:** Hydrogen-bond geometry (Å, °)

*D*—H⋯*A*	*D*—H	H⋯*A*	*D*⋯*A*	*D*—H⋯*A*
N4—H4*A*⋯O1^i^	0.88	2.44	3.266 (3)	157
N4—H4*B*⋯O2^ii^	0.88	2.30	3.108 (4)	152
N8—H8*A*⋯O3^iii^	0.88	2.54	3.084 (3)	120
N8—H8*B*⋯O5^iv^	0.88	2.26	3.114 (4)	162
O5—H5⋯O4^v^	0.89 (4)	1.91 (4)	2.785 (3)	169 (4)

Symmetry codes: (i) 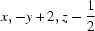; (ii) 
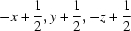
; (iii) 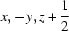; (iv) 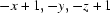; (v) 
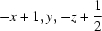
.

**Fig. 1.**
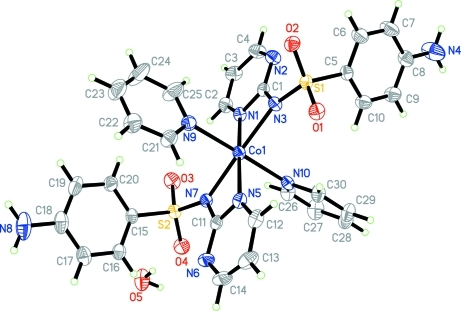
The molecular structure, showing the atom-labelling scheme. Displacement ellipsoids are drawn at the 50% probability level.

**Fig. 2.**
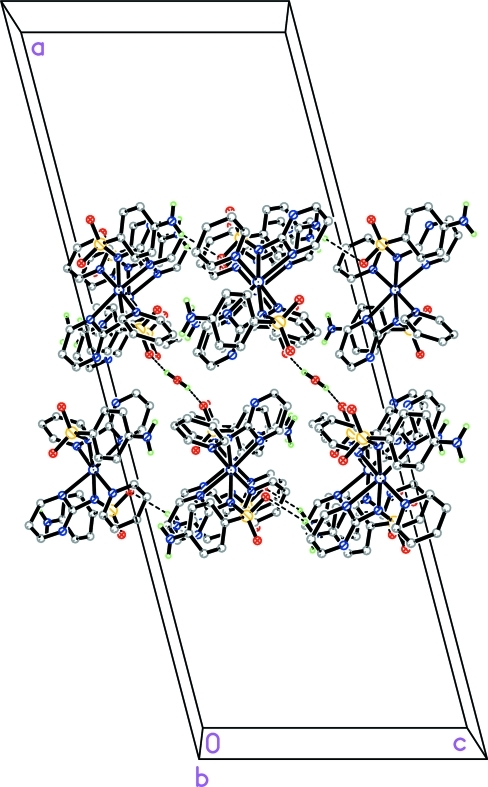
A packing plot of the title compound view along [010]. Hydrogen bonds are shown in the dashingline, and the H atoms that are not involved in hydrogen bonding are omitted.

## supplementary crystallographic information

### Comment

The title compound consists of [Co(C_11_H_11_N_4_O_4_S_2_] and half a lattice water molecule and is isostructural with trans-[Ni(sdz)_2_(py)_2_] (where sdz = sulfadiazine anion and py = pyridine)(Wang *et al.*, 2009), the title cobalt(II) complex has six-coordinate distorted octahedral geometry and contains two bidentate N-coordinated sulfadiazinate anion and two pyridine molecules occupying the trans sites. One water molecule lies on a 2-fold rotation axis. The coordination mode of sulfadiazine is similar to its cobalt(II) complex (Ajibade *et al.*, 2006) and copper(II) complex (Brown *et al.*, 1987), but different from Zn(sdz)_2_ (Yuan *et al.*, 2001), polymeric Cd(II) complex (Wang *et al.* 2005), and its copper complex (Hossain *et al.* 2006). The Co—N bond distances involving the sulfonamide atoms N3, N7, the pyrimido atoms N1, N5, and the pyridine atoms N9, N10, are very similar, at 2.132 (2), 2.091 (2), 2.124 (2), 2.168 (2), 2.196 (2), 2.193 (2) Å, respectively. The tetrahedral coordination at S is distorted, as is also found in the neutral sulfadiazine molecule. A three dimensional network is generated by N—H···O and O—H···O hydrogen bonds involving the complex and water molecules.

### Experimental

0.1 mmol Co(CH_3_COO)_2_.3H_2_O, 0.2 mmol sulfadiazine, ethanol (2.2 ml), water (0.2 ml) and pyridine (0.2 ml) were placed in a Pyrex tube (ca 25 cm). The tube was frozen with liquid N_2_, evacuated under vacuum, sealed with a torch and heated at 353 K for three days to give red-brown block-shaped crystals, with a yield of 70%.

### Refinement

The water H were found in a difference Fourier map and refined freely. Other H atoms were treated as riding, with C—H distances of 0.95 Å,*N*—H distances of 0.88 Å, were refined as riding with U_iso_(H) = 1.2U_eq_(C,N).

### Crystal data

**Table:**

[Co(C_10_H_9_N_4_O_2_S)_2_(C_5_H_5_N)_2_]·0.5H_2_O	*F*(000) = 2992
*M**_r_* = 724.68	*D*_x_ = 1.534 Mg m^−^^3^
Monoclinic, *C*2/*c*	Mo *K*α radiation, λ = 0.71070 Å
Hall symbol: -C 2yc	Cell parameters from 11616 reflections
*a* = 39.618 (4) Å	θ = 3.0–27.5°
*b* = 11.2407 (9) Å	µ = 0.74 mm^−^^1^
*c* = 14.5673 (13) Å	*T* = 193 K
β = 104.648 (2)°	Block, red-brown
*V* = 6276.4 (10) Å^3^	0.44 × 0.15 × 0.12 mm
*Z* = 8	

### Data collection

**Table:**

Rigaku Mercury diffractometer	7188 independent reflections
Radiation source: fine-focus sealed tube	6038 reflections with *I* > 2σ(*I*)
graphite	*R*_int_ = 0.051
Detector resolution: 7.31 pixels mm^-1^	θ_max_ = 27.5°, θ_min_ = 3.0°
ω scans	*h* = −51→51
Absorption correction: multi-scan (REQAB; Jacobson, 1998)	*k* = −12→14
*T*_min_ = 0.738, *T*_max_ = 0.917	*l* = −18→15
34520 measured reflections	

### Refinement

**Table:**

Refinement on *F*^2^	Primary atom site location: structure-invariant direct methods
Least-squares matrix: full	Secondary atom site location: difference Fourier map
*R*[*F*^2^ > 2σ(*F*^2^)] = 0.055	Hydrogen site location: inferred from neighbouring sites
*wR*(*F*^2^) = 0.103	H atoms treated by a mixture of independent and constrained refinement
*S* = 1.18	*w* = 1/[σ^2^(*F*_o_^2^) + (0.027*P*)^2^ + 11.6654*P*] where *P* = (*F*_o_^2^ + 2*F*_c_^2^)/3
7188 reflections	(Δ/σ)_max_ = 0.001
434 parameters	Δρ_max_ = 0.36 e Å^−^^3^
0 restraints	Δρ_min_ = −0.52 e Å^−^^3^

### Special details

**Table:**

Geometry. All esds (except the esd in the dihedral angle between two l.s. planes) are estimated using the full covariance matrix. The cell esds are taken into account individually in the estimation of esds in distances, angles and torsion angles; correlations between esds in cell parameters are only used when they are defined by crystal symmetry. An approximate (isotropic) treatment of cell esds is used for estimating esds involving l.s. planes.
Refinement. Refinement of *F*^2^ against ALL reflections. The weighted *R*-factor wR and goodness of fit *S* are based on *F*^2^, conventional *R*-factors *R* are based on *F*, with *F* set to zero for negative *F*^2^. The threshold expression of *F*^2^ > σ(*F*^2^) is used only for calculating *R*-factors(gt) etc. and is not relevant to the choice of reflections for refinement. *R*-factors based on *F*^2^ are statistically about twice as large as those based on *F*, and *R*- factors based on ALL data will be even larger.

### Fractional atomic coordinates and isotropic or equivalent isotropic displacement parameters (Å^2^)

**Table:**

	*x*	*y*	*z*	*U*_iso_*/*U*_eq_	
Co1	0.376102 (9)	0.51879 (3)	0.36763 (2)	0.02249 (10)	
S1	0.316541 (17)	0.74751 (6)	0.38330 (4)	0.02182 (14)	
S2	0.428760 (18)	0.28343 (6)	0.33767 (4)	0.02344 (15)	
O1	0.34287 (5)	0.78002 (16)	0.46799 (12)	0.0283 (4)	
O2	0.28292 (5)	0.71697 (16)	0.39809 (13)	0.0278 (4)	
O3	0.40112 (5)	0.26443 (17)	0.25308 (13)	0.0339 (5)	
O4	0.46383 (5)	0.28960 (17)	0.32502 (14)	0.0327 (5)	
O5	0.5000	0.1180 (3)	0.2500	0.0491 (9)	
N1	0.34102 (6)	0.50839 (19)	0.23097 (15)	0.0232 (5)	
N2	0.28923 (6)	0.6275 (2)	0.18964 (16)	0.0284 (5)	
N3	0.33361 (6)	0.64112 (19)	0.33688 (15)	0.0230 (5)	
N4	0.29295 (8)	1.1427 (3)	0.1053 (2)	0.0640 (10)	
H4A	0.3103	1.1712	0.0844	0.077*	
H4B	0.2718	1.1724	0.0845	0.077*	
N5	0.41670 (6)	0.53040 (19)	0.49884 (15)	0.0235 (5)	
N6	0.46858 (6)	0.4129 (2)	0.51736 (16)	0.0274 (5)	
N7	0.41832 (5)	0.40206 (18)	0.38452 (14)	0.0212 (5)	
N8	0.42558 (9)	−0.0979 (2)	0.6145 (2)	0.0587 (9)	
H8A	0.4058	−0.1331	0.6145	0.070*	
H8B	0.4449	−0.1194	0.6557	0.070*	
N9	0.34589 (6)	0.3841 (2)	0.42168 (16)	0.0274 (5)	
N10	0.40774 (6)	0.65618 (19)	0.32242 (15)	0.0239 (5)	
C1	0.31931 (7)	0.5951 (2)	0.24867 (18)	0.0229 (5)	
C2	0.33096 (8)	0.4491 (2)	0.14931 (19)	0.0298 (6)	
H2	0.3454	0.3875	0.1357	0.036*	
C3	0.30007 (8)	0.4755 (3)	0.0844 (2)	0.0339 (7)	
H3	0.2927	0.4334	0.0262	0.041*	
C4	0.28045 (8)	0.5663 (3)	0.1082 (2)	0.0344 (7)	
H4	0.2593	0.5868	0.0638	0.041*	
C5	0.30980 (7)	0.8664 (2)	0.30240 (18)	0.0239 (6)	
C6	0.27690 (8)	0.9142 (3)	0.2684 (2)	0.0373 (7)	
H6	0.2579	0.8836	0.2900	0.045*	
C7	0.27134 (8)	1.0056 (3)	0.2037 (2)	0.0443 (8)	
H7	0.2486	1.0378	0.1813	0.053*	
C8	0.29878 (8)	1.0518 (3)	0.1703 (2)	0.0342 (7)	
C9	0.33169 (8)	1.0030 (3)	0.2045 (2)	0.0348 (7)	
H9	0.3507	1.0330	0.1827	0.042*	
C10	0.33721 (7)	0.9114 (3)	0.2697 (2)	0.0318 (6)	
H10	0.3599	0.8789	0.2924	0.038*	
C11	0.43653 (7)	0.4463 (2)	0.47028 (17)	0.0224 (5)	
C12	0.43111 (8)	0.5883 (2)	0.57944 (19)	0.0296 (6)	
H12	0.4181	0.6479	0.6014	0.036*	
C13	0.46446 (8)	0.5632 (3)	0.6311 (2)	0.0353 (7)	
H13	0.4750	0.6054	0.6876	0.042*	
C14	0.48192 (7)	0.4741 (3)	0.5973 (2)	0.0335 (7)	
H14	0.5048	0.4549	0.6329	0.040*	
C15	0.42831 (7)	0.1675 (2)	0.41761 (18)	0.0235 (5)	
C16	0.45886 (7)	0.1330 (2)	0.48288 (19)	0.0267 (6)	
H16	0.4804	0.1699	0.4823	0.032*	
C17	0.45791 (8)	0.0452 (2)	0.5484 (2)	0.0336 (7)	
H17	0.4788	0.0216	0.5926	0.040*	
C18	0.42632 (9)	−0.0092 (3)	0.5502 (2)	0.0376 (7)	
C19	0.39586 (9)	0.0261 (3)	0.4843 (2)	0.0381 (7)	
H19	0.3743	−0.0107	0.4845	0.046*	
C20	0.39676 (8)	0.1139 (2)	0.4190 (2)	0.0311 (6)	
H20	0.3759	0.1379	0.3748	0.037*	
C21	0.35814 (8)	0.3409 (3)	0.5089 (2)	0.0326 (7)	
H21	0.3788	0.3751	0.5475	0.039*	
C22	0.34273 (8)	0.2498 (3)	0.5466 (2)	0.0389 (7)	
H22	0.3526	0.2225	0.6094	0.047*	
C23	0.31292 (9)	0.1993 (3)	0.4922 (3)	0.0495 (9)	
H23	0.3017	0.1359	0.5161	0.059*	
C24	0.29964 (10)	0.2425 (4)	0.4023 (3)	0.0661 (12)	
H24	0.2790	0.2096	0.3626	0.079*	
C25	0.31661 (9)	0.3340 (3)	0.3703 (2)	0.0515 (9)	
H25	0.3070	0.3635	0.3081	0.062*	
C26	0.41834 (8)	0.6433 (3)	0.2431 (2)	0.0336 (7)	
H26	0.4118	0.5732	0.2064	0.040*	
C27	0.43829 (9)	0.7266 (3)	0.2117 (2)	0.0426 (8)	
H27	0.4450	0.7142	0.1542	0.051*	
C28	0.44845 (8)	0.8276 (3)	0.2641 (2)	0.0359 (7)	
H28	0.4623	0.8860	0.2438	0.043*	
C29	0.43811 (8)	0.8426 (3)	0.3469 (2)	0.0346 (7)	
H29	0.4448	0.9113	0.3852	0.041*	
C30	0.41771 (8)	0.7553 (2)	0.3730 (2)	0.0307 (6)	
H30	0.4104	0.7662	0.4297	0.037*	
H5	0.5105 (11)	0.168 (4)	0.219 (3)	0.082 (15)*	

### Atomic displacement parameters (Å^2^)

**Table:**

	*U*^11^	*U*^22^	*U*^33^	*U*^12^	*U*^13^	*U*^23^
Co1	0.0261 (2)	0.02007 (19)	0.02013 (18)	0.00081 (14)	0.00363 (14)	−0.00073 (14)
S1	0.0264 (3)	0.0205 (3)	0.0191 (3)	−0.0007 (3)	0.0067 (3)	−0.0014 (2)
S2	0.0288 (4)	0.0227 (3)	0.0198 (3)	0.0009 (3)	0.0078 (3)	−0.0013 (2)
O1	0.0332 (11)	0.0287 (10)	0.0203 (9)	−0.0015 (8)	0.0018 (8)	−0.0045 (8)
O2	0.0289 (10)	0.0292 (10)	0.0283 (10)	−0.0026 (8)	0.0126 (8)	0.0016 (8)
O3	0.0420 (12)	0.0340 (11)	0.0222 (10)	0.0031 (9)	0.0015 (9)	−0.0070 (8)
O4	0.0334 (11)	0.0361 (12)	0.0338 (11)	0.0020 (9)	0.0182 (9)	0.0035 (9)
O5	0.070 (3)	0.0337 (19)	0.054 (2)	0.000	0.034 (2)	0.000
N1	0.0275 (12)	0.0222 (11)	0.0201 (11)	0.0008 (9)	0.0064 (9)	−0.0005 (9)
N2	0.0277 (12)	0.0318 (13)	0.0233 (12)	0.0029 (10)	0.0017 (10)	−0.0038 (10)
N3	0.0259 (12)	0.0223 (12)	0.0195 (11)	0.0035 (9)	0.0035 (9)	−0.0014 (9)
N4	0.0510 (19)	0.072 (2)	0.075 (2)	0.0204 (16)	0.0289 (17)	0.0521 (19)
N5	0.0290 (12)	0.0236 (12)	0.0182 (11)	−0.0032 (9)	0.0064 (9)	−0.0018 (9)
N6	0.0228 (12)	0.0341 (13)	0.0235 (12)	−0.0022 (10)	0.0028 (9)	0.0009 (10)
N7	0.0240 (12)	0.0194 (11)	0.0191 (11)	−0.0002 (8)	0.0033 (9)	−0.0022 (8)
N8	0.089 (3)	0.0385 (17)	0.0559 (19)	−0.0041 (16)	0.0324 (18)	0.0179 (14)
N9	0.0274 (12)	0.0271 (13)	0.0289 (12)	0.0007 (9)	0.0092 (10)	0.0007 (10)
N10	0.0273 (12)	0.0226 (12)	0.0214 (11)	0.0050 (9)	0.0052 (9)	0.0018 (9)
C1	0.0263 (14)	0.0207 (13)	0.0222 (13)	−0.0023 (10)	0.0071 (11)	0.0017 (10)
C2	0.0361 (16)	0.0270 (15)	0.0286 (15)	0.0002 (12)	0.0126 (13)	−0.0068 (12)
C3	0.0321 (16)	0.0412 (17)	0.0257 (14)	−0.0037 (13)	0.0026 (12)	−0.0109 (13)
C4	0.0295 (16)	0.0438 (18)	0.0266 (15)	0.0023 (13)	0.0010 (12)	−0.0039 (13)
C5	0.0282 (14)	0.0221 (14)	0.0211 (13)	0.0010 (11)	0.0058 (11)	0.0005 (10)
C6	0.0324 (16)	0.0361 (17)	0.0483 (19)	0.0067 (13)	0.0192 (15)	0.0154 (14)
C7	0.0334 (17)	0.047 (2)	0.056 (2)	0.0150 (14)	0.0176 (15)	0.0235 (16)
C8	0.0379 (17)	0.0331 (16)	0.0338 (16)	0.0054 (13)	0.0135 (13)	0.0102 (13)
C9	0.0321 (16)	0.0403 (18)	0.0347 (16)	−0.0015 (13)	0.0133 (13)	0.0103 (13)
C10	0.0256 (15)	0.0366 (17)	0.0329 (16)	0.0025 (12)	0.0068 (12)	0.0068 (13)
C11	0.0270 (14)	0.0214 (13)	0.0196 (12)	−0.0045 (10)	0.0075 (11)	0.0014 (10)
C12	0.0392 (17)	0.0283 (15)	0.0237 (14)	−0.0078 (12)	0.0124 (12)	−0.0038 (11)
C13	0.0368 (17)	0.0457 (18)	0.0219 (14)	−0.0129 (14)	0.0045 (13)	−0.0094 (13)
C14	0.0250 (15)	0.0484 (18)	0.0240 (14)	−0.0066 (13)	0.0005 (12)	0.0008 (13)
C15	0.0296 (14)	0.0183 (13)	0.0241 (13)	0.0004 (10)	0.0094 (11)	−0.0022 (10)
C16	0.0327 (15)	0.0227 (14)	0.0261 (14)	−0.0010 (11)	0.0099 (12)	−0.0024 (11)
C17	0.0467 (19)	0.0267 (15)	0.0267 (15)	0.0021 (13)	0.0078 (13)	−0.0005 (12)
C18	0.061 (2)	0.0236 (15)	0.0347 (17)	0.0010 (14)	0.0249 (16)	−0.0014 (12)
C19	0.0451 (19)	0.0239 (15)	0.054 (2)	−0.0077 (13)	0.0279 (16)	−0.0054 (14)
C20	0.0302 (15)	0.0237 (14)	0.0411 (17)	−0.0003 (11)	0.0120 (13)	−0.0040 (12)
C21	0.0344 (16)	0.0353 (17)	0.0283 (15)	−0.0069 (13)	0.0083 (13)	0.0007 (12)
C22	0.0427 (18)	0.0438 (18)	0.0321 (16)	−0.0025 (14)	0.0130 (14)	0.0088 (14)
C23	0.048 (2)	0.050 (2)	0.050 (2)	−0.0153 (16)	0.0130 (17)	0.0136 (17)
C24	0.054 (2)	0.082 (3)	0.053 (2)	−0.042 (2)	−0.0043 (19)	0.023 (2)
C25	0.040 (2)	0.068 (2)	0.0388 (19)	−0.0234 (17)	−0.0046 (15)	0.0137 (17)
C26	0.0466 (19)	0.0306 (16)	0.0249 (15)	−0.0012 (13)	0.0116 (13)	−0.0042 (12)
C27	0.062 (2)	0.0409 (19)	0.0319 (17)	−0.0024 (16)	0.0254 (16)	−0.0008 (14)
C28	0.0407 (18)	0.0327 (16)	0.0383 (17)	−0.0059 (13)	0.0174 (14)	0.0024 (13)
C29	0.0436 (18)	0.0268 (16)	0.0346 (16)	−0.0079 (13)	0.0123 (14)	−0.0055 (12)
C30	0.0416 (17)	0.0259 (15)	0.0271 (15)	−0.0017 (12)	0.0135 (13)	−0.0041 (11)

### Geometric parameters (Å, °)

**Table:**

Co1—N7	2.091 (2)	C6—C7	1.374 (4)
Co1—N1	2.124 (2)	C6—H6	0.9500
Co1—N3	2.132 (2)	C7—C8	1.398 (4)
Co1—N5	2.168 (2)	C7—H7	0.9500
Co1—N10	2.193 (2)	C8—C9	1.386 (4)
Co1—N9	2.196 (2)	C9—C10	1.381 (4)
S1—O2	1.4436 (19)	C9—H9	0.9500
S1—O1	1.4466 (19)	C10—H10	0.9500
S1—N3	1.605 (2)	C12—C13	1.375 (4)
S1—C5	1.757 (3)	C12—H12	0.9500
S2—O3	1.443 (2)	C13—C14	1.376 (4)
S2—O4	1.449 (2)	C13—H13	0.9500
S2—N7	1.599 (2)	C14—H14	0.9500
S2—C15	1.751 (3)	C15—C16	1.392 (4)
O5—H5	0.89 (4)	C15—C20	1.392 (4)
N1—C2	1.334 (3)	C16—C17	1.380 (4)
N1—C1	1.366 (3)	C16—H16	0.9500
N2—C1	1.332 (3)	C17—C18	1.399 (4)
N2—C4	1.339 (3)	C17—H17	0.9500
N3—C1	1.368 (3)	C18—C19	1.396 (5)
N4—C8	1.373 (4)	C19—C20	1.378 (4)
N4—H4A	0.8800	C19—H19	0.9500
N4—H4B	0.8800	C20—H20	0.9500
N5—C12	1.338 (3)	C21—C22	1.376 (4)
N5—C11	1.360 (3)	C21—H21	0.9500
N6—C11	1.335 (3)	C22—C23	1.368 (5)
N6—C14	1.342 (3)	C22—H22	0.9500
N7—C11	1.368 (3)	C23—C24	1.372 (5)
N8—C18	1.373 (4)	C23—H23	0.9500
N8—H8A	0.8800	C24—C25	1.373 (5)
N8—H8B	0.8800	C24—H24	0.9500
N9—C21	1.332 (4)	C25—H25	0.9500
N9—C25	1.335 (4)	C26—C27	1.376 (4)
N10—C26	1.334 (3)	C26—H26	0.9500
N10—C30	1.339 (3)	C27—C28	1.371 (4)
C2—C3	1.376 (4)	C27—H27	0.9500
C2—H2	0.9500	C28—C29	1.379 (4)
C3—C4	1.379 (4)	C28—H28	0.9500
C3—H3	0.9500	C29—C30	1.385 (4)
C4—H4	0.9500	C29—H29	0.9500
C5—C6	1.380 (4)	C30—H30	0.9500
C5—C10	1.386 (4)		
			
N7—Co1—N1	112.97 (8)	C6—C7—H7	119.6
N7—Co1—N3	174.43 (8)	C8—C7—H7	119.6
N1—Co1—N3	62.84 (8)	N4—C8—C9	121.4 (3)
N7—Co1—N5	62.69 (8)	N4—C8—C7	120.4 (3)
N1—Co1—N5	173.41 (8)	C9—C8—C7	118.2 (3)
N3—Co1—N5	121.11 (8)	C10—C9—C8	120.9 (3)
N7—Co1—N10	88.64 (8)	C10—C9—H9	119.6
N1—Co1—N10	92.61 (8)	C8—C9—H9	119.6
N3—Co1—N10	87.91 (8)	C9—C10—C5	120.4 (3)
N5—Co1—N10	82.50 (8)	C9—C10—H10	119.8
N7—Co1—N9	91.00 (8)	C5—C10—H10	119.8
N1—Co1—N9	90.65 (8)	N6—C11—N5	125.9 (2)
N3—Co1—N9	92.70 (8)	N6—C11—N7	125.5 (2)
N5—Co1—N9	94.33 (8)	N5—C11—N7	108.6 (2)
N10—Co1—N9	176.60 (8)	N5—C12—C13	121.3 (3)
O2—S1—O1	115.23 (11)	N5—C12—H12	119.4
O2—S1—N3	112.88 (11)	C13—C12—H12	119.4
O1—S1—N3	104.94 (11)	C12—C13—C14	117.0 (3)
O2—S1—C5	106.95 (12)	C12—C13—H13	121.5
O1—S1—C5	109.53 (12)	C14—C13—H13	121.5
N3—S1—C5	107.05 (12)	N6—C14—C13	124.1 (3)
O3—S2—O4	116.35 (12)	N6—C14—H14	117.9
O3—S2—N7	105.41 (11)	C13—C14—H14	117.9
O4—S2—N7	111.97 (12)	C16—C15—C20	119.9 (3)
O3—S2—C15	108.95 (12)	C16—C15—S2	120.6 (2)
O4—S2—C15	106.96 (12)	C20—C15—S2	119.5 (2)
N7—S2—C15	106.82 (11)	C17—C16—C15	120.0 (3)
C2—N1—C1	117.7 (2)	C17—C16—H16	120.0
C2—N1—Co1	147.23 (19)	C15—C16—H16	120.0
C1—N1—Co1	94.47 (15)	C16—C17—C18	120.4 (3)
C1—N2—C4	114.9 (2)	C16—C17—H17	119.8
C1—N3—S1	123.99 (18)	C18—C17—H17	119.8
C1—N3—Co1	94.06 (15)	N8—C18—C19	120.7 (3)
S1—N3—Co1	141.87 (13)	N8—C18—C17	120.2 (3)
C8—N4—H4A	120.0	C19—C18—C17	119.0 (3)
C8—N4—H4B	120.0	C20—C19—C18	120.6 (3)
H4A—N4—H4B	120.0	C20—C19—H19	119.7
C12—N5—C11	117.1 (2)	C18—C19—H19	119.7
C12—N5—Co1	149.3 (2)	C19—C20—C15	120.0 (3)
C11—N5—Co1	92.62 (15)	C19—C20—H20	120.0
C11—N6—C14	114.6 (2)	C15—C20—H20	120.0
C11—N7—S2	123.98 (18)	N9—C21—C22	124.1 (3)
C11—N7—Co1	95.79 (16)	N9—C21—H21	117.9
S2—N7—Co1	139.33 (12)	C22—C21—H21	117.9
C18—N8—H8A	120.0	C23—C22—C21	118.9 (3)
C18—N8—H8B	120.0	C23—C22—H22	120.6
H8A—N8—H8B	120.0	C21—C22—H22	120.6
C21—N9—C25	115.9 (3)	C22—C23—C24	118.2 (3)
C21—N9—Co1	119.55 (19)	C22—C23—H23	120.9
C25—N9—Co1	124.4 (2)	C24—C23—H23	120.9
C26—N10—C30	117.0 (2)	C23—C24—C25	119.1 (3)
C26—N10—Co1	120.87 (18)	C23—C24—H24	120.4
C30—N10—Co1	122.14 (18)	C25—C24—H24	120.4
N2—C1—N1	125.2 (2)	N9—C25—C24	123.8 (3)
N2—C1—N3	126.4 (2)	N9—C25—H25	118.1
N1—C1—N3	108.4 (2)	C24—C25—H25	118.1
N1—C2—C3	121.2 (3)	N10—C26—C27	123.2 (3)
N1—C2—H2	119.4	N10—C26—H26	118.4
C3—C2—H2	119.4	C27—C26—H26	118.4
C2—C3—C4	116.6 (3)	C28—C27—C26	119.4 (3)
C2—C3—H3	121.7	C28—C27—H27	120.3
C4—C3—H3	121.7	C26—C27—H27	120.3
N2—C4—C3	124.5 (3)	C27—C28—C29	118.6 (3)
N2—C4—H4	117.8	C27—C28—H28	120.7
C3—C4—H4	117.8	C29—C28—H28	120.7
C6—C5—C10	119.0 (3)	C28—C29—C30	118.5 (3)
C6—C5—S1	120.4 (2)	C28—C29—H29	120.8
C10—C5—S1	120.5 (2)	C30—C29—H29	120.8
C7—C6—C5	120.7 (3)	N10—C30—C29	123.4 (3)
C7—C6—H6	119.7	N10—C30—H30	118.3
C5—C6—H6	119.7	C29—C30—H30	118.3
C6—C7—C8	120.8 (3)		
			
N7—Co1—N1—C2	12.2 (4)	Co1—N3—C1—N1	−4.2 (2)
N3—Co1—N1—C2	−171.9 (4)	C1—N1—C2—C3	1.1 (4)
N10—Co1—N1—C2	101.8 (3)	Co1—N1—C2—C3	168.7 (2)
N9—Co1—N1—C2	−79.2 (3)	N1—C2—C3—C4	0.4 (4)
N7—Co1—N1—C1	−178.81 (14)	C1—N2—C4—C3	0.4 (4)
N3—Co1—N1—C1	−2.87 (14)	C2—C3—C4—N2	−1.1 (5)
N10—Co1—N1—C1	−89.18 (15)	O2—S1—C5—C6	1.9 (3)
N9—Co1—N1—C1	89.83 (15)	O1—S1—C5—C6	−123.6 (2)
O2—S1—N3—C1	63.1 (2)	N3—S1—C5—C6	123.1 (2)
O1—S1—N3—C1	−170.7 (2)	O2—S1—C5—C10	−176.3 (2)
C5—S1—N3—C1	−54.3 (2)	O1—S1—C5—C10	58.2 (3)
O2—S1—N3—Co1	−112.6 (2)	N3—S1—C5—C10	−55.0 (3)
O1—S1—N3—Co1	13.6 (2)	C10—C5—C6—C7	−0.5 (5)
C5—S1—N3—Co1	130.0 (2)	S1—C5—C6—C7	−178.7 (3)
N1—Co1—N3—C1	2.87 (14)	C5—C6—C7—C8	0.4 (5)
N5—Co1—N3—C1	176.82 (14)	C6—C7—C8—N4	179.7 (3)
N10—Co1—N3—C1	96.88 (15)	C6—C7—C8—C9	−0.1 (5)
N9—Co1—N3—C1	−86.47 (16)	N4—C8—C9—C10	−179.8 (3)
N1—Co1—N3—S1	179.3 (2)	C7—C8—C9—C10	−0.1 (5)
N5—Co1—N3—S1	−6.7 (2)	C8—C9—C10—C5	−0.1 (5)
N10—Co1—N3—S1	−86.7 (2)	C6—C5—C10—C9	0.3 (4)
N9—Co1—N3—S1	90.0 (2)	S1—C5—C10—C9	178.5 (2)
N7—Co1—N5—C12	169.1 (4)	C14—N6—C11—N5	−3.2 (4)
N3—Co1—N5—C12	−6.3 (4)	C14—N6—C11—N7	176.1 (2)
N10—Co1—N5—C12	76.7 (4)	C12—N5—C11—N6	2.9 (4)
N9—Co1—N5—C12	−102.0 (4)	Co1—N5—C11—N6	174.8 (2)
N7—Co1—N5—C11	3.19 (14)	C12—N5—C11—N7	−176.5 (2)
N3—Co1—N5—C11	−172.13 (14)	Co1—N5—C11—N7	−4.6 (2)
N10—Co1—N5—C11	−89.16 (15)	S2—N7—C11—N6	14.3 (4)
N9—Co1—N5—C11	92.08 (15)	Co1—N7—C11—N6	−174.6 (2)
O3—S2—N7—C11	174.9 (2)	S2—N7—C11—N5	−166.26 (17)
O4—S2—N7—C11	−57.6 (2)	Co1—N7—C11—N5	4.8 (2)
C15—S2—N7—C11	59.1 (2)	C11—N5—C12—C13	−0.3 (4)
O3—S2—N7—Co1	8.7 (2)	Co1—N5—C12—C13	−164.4 (3)
O4—S2—N7—Co1	136.13 (18)	N5—C12—C13—C14	−1.5 (4)
C15—S2—N7—Co1	−107.1 (2)	C11—N6—C14—C13	1.2 (4)
N1—Co1—N7—C11	171.33 (14)	C12—C13—C14—N6	1.0 (4)
N5—Co1—N7—C11	−3.19 (14)	O3—S2—C15—C16	151.9 (2)
N10—Co1—N7—C11	79.07 (15)	O4—S2—C15—C16	25.3 (2)
N9—Co1—N7—C11	−97.55 (15)	N7—S2—C15—C16	−94.7 (2)
N1—Co1—N7—S2	−20.1 (2)	O3—S2—C15—C20	−31.5 (2)
N5—Co1—N7—S2	165.4 (2)	O4—S2—C15—C20	−158.0 (2)
N10—Co1—N7—S2	−112.37 (19)	N7—S2—C15—C20	81.9 (2)
N9—Co1—N7—S2	71.01 (19)	C20—C15—C16—C17	0.4 (4)
N7—Co1—N9—C21	59.8 (2)	S2—C15—C16—C17	177.0 (2)
N1—Co1—N9—C21	172.8 (2)	C15—C16—C17—C18	−0.4 (4)
N3—Co1—N9—C21	−124.3 (2)	C16—C17—C18—N8	178.9 (3)
N5—Co1—N9—C21	−2.9 (2)	C16—C17—C18—C19	0.4 (4)
N7—Co1—N9—C25	−114.9 (3)	N8—C18—C19—C20	−179.1 (3)
N1—Co1—N9—C25	−1.9 (3)	C17—C18—C19—C20	−0.5 (4)
N3—Co1—N9—C25	60.9 (3)	C18—C19—C20—C15	0.6 (4)
N5—Co1—N9—C25	−177.6 (3)	C16—C15—C20—C19	−0.6 (4)
N7—Co1—N10—C26	64.3 (2)	S2—C15—C20—C19	−177.2 (2)
N1—Co1—N10—C26	−48.6 (2)	C25—N9—C21—C22	0.6 (5)
N3—Co1—N10—C26	−111.3 (2)	Co1—N9—C21—C22	−174.6 (2)
N5—Co1—N10—C26	127.0 (2)	N9—C21—C22—C23	0.0 (5)
N7—Co1—N10—C30	−114.7 (2)	C21—C22—C23—C24	−0.4 (6)
N1—Co1—N10—C30	132.3 (2)	C22—C23—C24—C25	0.1 (6)
N3—Co1—N10—C30	69.6 (2)	C21—N9—C25—C24	−0.9 (6)
N5—Co1—N10—C30	−52.1 (2)	Co1—N9—C25—C24	174.0 (3)
C4—N2—C1—N1	1.2 (4)	C23—C24—C25—N9	0.6 (7)
C4—N2—C1—N3	−178.2 (3)	C30—N10—C26—C27	−0.7 (4)
C2—N1—C1—N2	−1.9 (4)	Co1—N10—C26—C27	−179.8 (2)
Co1—N1—C1—N2	−175.3 (2)	N10—C26—C27—C28	0.9 (5)
C2—N1—C1—N3	177.5 (2)	C26—C27—C28—C29	−0.3 (5)
Co1—N1—C1—N3	4.2 (2)	C27—C28—C29—C30	−0.4 (5)
S1—N3—C1—N2	−2.1 (4)	C26—N10—C30—C29	−0.1 (4)
Co1—N3—C1—N2	175.3 (2)	Co1—N10—C30—C29	179.0 (2)
S1—N3—C1—N1	178.46 (17)	C28—C29—C30—N10	0.7 (5)

### Hydrogen-bond geometry (Å, °)

**Table:**

*D*—H···*A*	*D*—H	H···*A*	*D*···*A*	*D*—H···*A*
N4—H4A···O1^i^	0.88	2.44	3.266 (3)	157
N4—H4B···O2^ii^	0.88	2.30	3.108 (4)	152
N8—H8A···O3^iii^	0.88	2.54	3.084 (3)	120
N8—H8B···O5^iv^	0.88	2.26	3.114 (4)	162
O5—H5···O4^v^	0.89 (4)	1.91 (4)	2.785 (3)	169 (4)

Symmetry codes: (i) *x*, −*y*+2, *z*−1/2; (ii) −*x*+1/2, *y*+1/2, −*z*+1/2; (iii) *x*, −*y*, *z*+1/2; (iv) −*x*+1, −*y*, −*z*+1; (v) −*x*+1, *y*, −*z*+1/2.
